# Undiagnosed cardiovascular risk factors including elevated lipoprotein(a) in patients with ischaemic heart disease

**DOI:** 10.3389/fepid.2023.1207752

**Published:** 2023-07-17

**Authors:** Fionn Chua, Audrey Lam, Ying Hui Mak, Zhong Hui Lee, Lily Mae Dacay, Jie Lin Yew, Troy Puar, Joan Khoo, Weien Chow, Vern Hsen Tan, Khim Leng Tong, Boon Wah Liew, Colin Yeo, Wann Jia Loh

**Affiliations:** ^1^ Dietetics Department, Changi General Hospital, Singapore, Singapore; ^2^ Department of Pharmacy, Changi General Hospital, Singapore, Singapore; ^3^ Department of Endocrinology, Changi General Hospital, Singapore, Singapore; ^4^ Department of Cardiology, Changi General Hospital, Singapore, Singapore; ^5^ Duke-NUS Medical School, Singapore, Singapore

**Keywords:** Ischaemic heart disease, diabetes, hypertension, hypercholesterolaemia, cardiovascular risk factors, Lp(a), lipoprotein(a), cardiovascular diet

## Abstract

**Objectives:**

This study aims to investigate the prevalence of undiagnosed cardiovascular risk factors in patients with ischaemic heart disease (IHD).

**Methods:**

We assessed the prevalence of previously undiagnosed cardiovascular risk factors, including elevated lipoprotein(a) [Lp(a)], among consenting patients with IHD who were admitted to hospital. Clinical information, including dietary history, from patients with newly diagnosed IHD and known IHD were compared.

**Results:**

Of the 555 patients, 82.3% were males and 48.5% of Chinese ethnicity. Overall, 13.3% were newly diagnosed with hypertension, 14.8% with hypercholesterolemia, and 5% with type 2 diabetes (T2DM). Patients with newly diagnosed IHD, compared to those with known IHD, had a higher prevalence of new diagnoses of hypercholesterolemia (29.1% vs. 2.0%, *p* < 0.001), hypertension (24.5% vs. 3.4%, *p* < 0.001) and T2DM (7.3% vs. 3.1%, *p* = 0.023). Active smoking was prevalent in 28.3% of patients, and higher in newly diagnosed IHD (34.1% vs. 23.2%, *p* = 0.005). Elevated Lp(a) of ≥120 nmol/L was detected in 15.6% of all patients, none of whom were previously diagnosed. Dietary habits of >50% of patients in both groups did not meet national recommendations for fruits, vegetables, wholegrain and oily fish intake. However, patients with known IHD had a more regular omega-3 supplement intake (23.4% vs. 10.3%, *p* = 0.024).

**Conclusion:**

Increased detection efforts is necessary to diagnose chronic metabolic diseases (hypertension, hypercholesterolemia, T2DM) especially among patients at high risk for IHD. Cardiovascular risk factors, in particular elevated Lp(a), smoking, and suboptimal dietary intake in patients with IHD deserve further attention.

## Introduction

Ischaemic heart disease (IHD) is a major cause of mortality and morbidity worldwide, accounting for 20.1% of all mortality in Singapore (1, 2). There were 11,631 reported cases of AMI in Singapore in the year 2020 alone (3). Risk factors for IHD include type 2 diabetes (T2DM), hypertension, hypercholesterolemia, obesity, smoking, alcohol consumption, physical inactivity and poor dietary habits (4). Another important cardiovascular risk factor is elevated lipoprotein(a) [Lp(a)] concentration. Recent studies and guidelines have highlighted that elevated Lp(a) is a prevalent causal risk factor for atherosclerotic cardiovascular disease (ASCVD) in the general population, which increases severity of ASCVD among patients with ASCVD (5–7). Multiple international guidelines have now recommended that Lp(a) be measured in all patients with ASCVD (5–7). However, elevated Lp(a) in patients with ASCVD remains underdiagnosed worldwide (8) and Lp(a) is not routinely measured locally.

The recent national population health survey (2019–2020) reported that the prevalence of T2DM, hypertension and hypercholesterolaemia had increased in recent years, with a current estimated prevalence of 9.5%, 35.5%, and 29.1% respectively (9). The increased prevalence may be partially contributed by the increased efforts, at the national level, in population screening for these cardiovascular risk factors e.g., Screen for Life and BEAT diabetes campaign (10). However, we observed from our clinical practice, that many patients were unaware of their co-existing metabolic comorbidities at the time of IHD diagnosis. Hence, this study aims to investigate whether the prevalence of cardiovascular risk factors, particularly those that are undiagnosed, is common among patients with known and newly diagnosed IHD.

## Methods

Patients with IHD who were admitted to Changi General Hospital (CGH) cardiology wards from 25th June 2020 to 31st December 2020 were recruited in our prospective study and consented for study participation. CGH is a 1,000-bed hospital in eastern Singapore serving more than a million people. There are approximately 1,000 patients per year admitted to cardiology wards for acute myocardial infarction. A total of 555 patients with IHD who were admitted to the hospital were recruited to the study. Among the eligible patients approached by us, there were 409 patients who declined to participate, and 318 patients were discharged before they could be recruited. Exclusion criteria for patient recruitment included lack of mental capacity and critically ill (e.g., cancer). This study was approved by our hospital ethics board.

All study participants were interviewed for comorbidities, smoking and alcohol history. From the electronic medical records, patient demographics, blood lipid profile, medications, co-morbidities, and dietary history were recorded. Obesity was defined as body mass index (BMI) ≥ 27.5 kg/m², as this is the cut-off point for obesity in Asians (10). Hypercholesterolemia was defined as highest ever low-density lipoprotein cholesterol concentration (LDL-C) ≥ 3.4 mmol/L (11). Newly diagnosed hypercholesterolemia was defined as patients with elevated LDL-C and without a past medical history of hypercholesterolaemia or hyperlipidaemia, or without the use of lipid lowering therapy. In patients who were already on statins, a correction factor was applied to LDL-C readings when pre-statin LDL was not available (12). Hyper-Lp(a) was defined as elevated Lp(a) levels of ≥120 nmol/L (5, 13).

The definition of healthy eating was as defined by the national healthy eating guidelines (14); (a) daily consumption of wholegrains, 2 serves of fruit and 2 serves of vegetables, (b) intake of eggs kept to a maximum of 3 per week, (c) consumption of oily fish at least twice a week. Low alcohol intake was defined as ≤1 standard drink per day for women and ≤2 standard drinks per day for men (14). Patients who were offered inpatient dietitian review had their diet history collected from the electronic medical records. As a dietitian referral is not compulsory for all patients in our study group, not all patients had been seen by a dietitian before, and some had missing fields in the diet record. Hence, the diet history for consumption of eggs, vegetables, fruits and wholegrain were available for approximately half of the group with known IHD and 60%–80% of the group with newly diagnosed IHD. The diet history for consumption of oily fish and omega-3 were available in approximately 30% of the group with known IHD and 45% of the group with newly diagnosed IHD.

Blood tests were taken and measured for plasma Lp(a) using particle-enhanced turbidimetric immunoassay with Tina-quant Lipoprotein(a) Gen.2 (Latex) Roche, and inter-assay coefficient of variation (CV) were ≤2.2%. Using an enzymatic colorimetry Roche Cobas c702 analyzer, the total cholesterol, high density lipoprotein cholesterol (HDL-C), direct LDL-C, and triglyceride levels were measured, and the inter-assay CVs were <1.5%. Variables were compared using Chi-square test for categorical data, results presented as %(*n*), and Mann-Whitney U test for continuous data, results presented as median (interquartile range). SPSS version 29.0 (IBM Corp., Armonk, United States) was used for statistical analyses. *P* value of <0.05 was taken as statistically significant.

## Results

Among the 555 study participants, 53% were known to have IHD, and 47% were newly diagnosed IHD. 82.3% were of male gender, and 48.5% were of Chinese ethnicity. The median BMI was 25.1 kg/m^2^, with 30.6% of study participants being obese (BMI ≥ 27.5 kg/m^2^). Patients with known IHD were generally older than patients with newly diagnosed IHD, with a median age of 67 years vs. 60 years old (*p* < 0.001). Overall, 13.3% of patients were newly diagnosed with hypertension, and 5% with T2DM. There was a significantly higher prevalence of newly diagnosed hypertension (24.5% vs. 3.4%, *p* < 0.001) and T2DM (7.3% vs. 3.1%, *p* = 0.023) in the group with newly diagnosed IHD compared to the group with known IHD. The prevalence of newly diagnosed hypercholesterolaemia with LDL-C ≥ 3.4 mmol/L was also significantly higher in patients with newly diagnosed IHD (29.1% vs. 2.0%, *p* < 0.001). Table 1 shows the comparison of these baseline characteristics of study participants between the 2 groups.

**Table 1:** Baseline characteristics of study participants, comparing patients with known IHD and newly diagnosed IHD.

	All Patients	Known IHD	Newly diagnosed IHD	*P* value
	(*n* = 555)	(*n* = 294)	(*n* = 261)	
Age (years)	63.8 (56.4, 71.0)	67.4 (60.0, 73.8)	60.1 (52.8, 68)	<0.001
BMI (kg/m^2^)	25.1 (22.4, 28.4)	25.0 (22.2, 28.8)	25.2 (22.6, 28.1)	0.797
BMI ≥ 27.5 kg/m^2^, %(*n*)	30.6 (169)	32.5 (95)	28.4 (74)	0.287
Gender (male), %(*n*)	82.3 (457)	81 (238)	83.9 (219)	0.362
Active smoking, %(*n*)	28.3 (157)	23.2 (68)	34.1 (89)	0.005
Ethnicity				
Chinese, %(*n*)	48.5 (269)	46.3 (136)	51 (133)	0.494
Malay, %(*n*)	32.3 (179)	35 (103)	29.1 (76)	
Indian, %(*n*)	12.6 (70)	11.9 (35)	13.4 (35)	
Others, %(*n*)	6.7 (37)	6.8 (20)	6.5 (17)	
Metabolic disease				
T2DM, %(*n*)	47.9 (266)	57.8 (170)	36.8 (96)	<0.001
New T2DM, %(*n*)	5 (28)	3.1 (9)	7.3 (19)	0.023
Hypertension, %(*n*)	79.3 (440)	85.4 (251)	72.4 (189)	<0.001
New Hypertension, %(*n*)	13.3 (74)	3.4 (10)	24.5 (64)	<0.001
Hypercholesterolaemia, %(*n*)	82.2 (456)	80.3 (236)	84.3 (220)	0.217
New Hypercholesterolaemia	14.8 (82)	2.0 (6)	29.1 (76)	<0.001
CKD, %(*n*)	24.5 (136)	35.4 (104)	12.3 (32)	<0.001
New CKD, %(*n*)	3.2 (18)	3.4 (10)	3.1 (8)	0.823
Laboratory diagnostic readings				
Total cholesterol (mmol/L)	4.02 (3.26, 5.05)	3.60 (3.02, 4.26)	4.52 (3.84, 5.60)	<0.001
LDL cholesterol (mmol/L)	2.40 (1.78, 3.43)	2.00 (1.55, 2.64)	3.08 (2.33, 4.13)	<0.001
HDL cholesterol (mmol/L)	1.13 (0.94, 1.33)	1.12 (0.92, 1.33)	1.14 (0.97, 1.37)	0.230
Triglyceride (mmol/L)	1.33 (0.96, 1.97)	1.30 (0.96, 2.00)	1.35 (0.97, 1.96)	0.916
Lp(a) (nmol/L)	35.2 (16.3, 80.4)	33.9 (15.1, 69.2)	40.6 (16.7, 87.8)	0.113
Lp(a) ≥ 120 nmol/L, %(*n*)	15.6 (81)	14.1 (39)	17.4 (42)	0.305

The percentage of all patients with metabolic disease comorbidities, as well as the subset of patients with newly diagnosed comorbidities, are presented. Values represented as median with interquartile ranges or % (*n*).

BMI, body mass index; T2DM, type 2 diabetes mellitus; CKD, chronic kidney disease; LDL, low density lipoprotein cholesterol; HDL, high density lipoprotein cholesterol; Lp(a), lipoprotein(a).

Elevated Lp(a) ≥ 120 nmol/L was present in 15.6% of all patients, with a median Lp(a) level of 35.2 nmol/L, no significant difference between the 2 groups. None of the study participants had previously had their Lp(a) level checked prior to this study. The prevalence of active smoking was 28.3% while ex-smokers were 22.6% among all the patients in this study. There were significantly more active smokers in the newly diagnosed IHD group compared to those with known IHD (34.1% vs. 23.2%, *p* = 0.005). The median total cholesterol (4.52 mmol/L vs. 3.6 mmol/L, *p* < 0.001) and direct LDL-C (3.08 mmol/L vs. 2.00 mmol/L, *p* < 0.001) were higher in the group of patients with newly diagnosed IHD compared to those with known IHD, reflecting the increased use of statin in patients with known IHD. Table 1 shows the laboratory diagnostic readings of study participants.

Overall, the dietary habits of >50% of patients whose diet histories were available did not meet national recommendations for fruits, vegetables, wholegrains, and oily fish intake. Dietary intake of study participants is shown in Table 2. Only ∼10% of patients took 2 serves of fruit daily, 20%–30% took 2 serves of vegetables daily, and ∼33% were wholegrain eaters. Majority of patients consumed alcohol within national guideline limits. The group with known IHD consumed significantly more regular omega-3 supplement intake than the group with newly diagnosed IHD (23.4% vs. 10.3%, *p* = 0.024), although there was no difference in oily fish intake. There was also no statistically significant difference in the percentage of patients meeting the recommended intake for eggs, vegetables, fruit, and wholegrains, between both groups.

**Table 2:** Dietary intake of study participants.

	All Patients	Known IHD	Newly diagnosed IHD	*p*-value
Low alcohol consumption, %(*n*)	96.9 (538)	97.3 (286)	96.6 (252)	0.620
Oily fish ≥2/week, %(*n*)	23.3 (48), *N* = 206	23.8 (20), *N* = 84	23 (28), *N* = 122	0.886
Omega-3 supplement intake, %(*n*)	15.5 (25), *N* = 161	23.4 (15), *N* = 64	10.3 (10), *N* = 97	0.024
Eggs ≤3/week, %(*n*)	50.4 (132), *N* = 262	54.6 (59), *N* = 108	47.4 (73), *N* = 154	0.249
Vegetables 2 serves/day, %(*n*)	24.8 (92), *N* = 371	19.7 (31), *N* = 157	28.5 (61), *N* = 214	0.054
Fruit 2 serves/day, %(*n*)	10.7 (35), *N* = 328	11.7 (15), *N* = 128	10 (20), *N* = 200	0.623
Wholegrain eaters, %(*n*)	34.2 (122), *N* = 357	30.8 (45), *N* = 146	36.5 (77), *N* = 211	0.267

As missing values were present for all dietary fields except alcohol consumption, the total subgroup sample size available for analysis were presented as *N* for individual dietary fields. Values represented as % (*n*).

## Discussion

Our study identified multiple important cardiovascular risk factors worthy to be considered for potential intervention to prevent ASCVD onset or progression in high-risk individuals. Firstly, we found that many patients with newly diagnosed IHD were previously not formally diagnosed or previously unaware of the diagnoses of these major cardiovascular comorbidities, namely hypertension, T2DM, hypercholesterolaemia, and hyper-Lp(a). As atherosclerosis requires years of exposure to risk factors, it is likely that these comorbidities could have been diagnosed years earlier through health screening (10, 15). Secondly, we report that none of the patients had previously had their Lp(a) level checked, and we identified 15.6% of patients with hyper-Lp(a). Individuals with elevated Lp(a) are at increased lifetime risk of ASCVD, with very elevated Lp(a) level of >430 nmol/L conferring the same risk for premature ASCVD as those with heterozygous familial hypercholesterolemia (7, 16). Despite recommendations by recent international guidelines that all ASCVD patients should have Lp(a) tested (13, 16–19) and that it is easily measurable by a blood test that does not require fasting, Lp(a) is currently not being routinely tested in Singapore. Similarly, other studies also report that elevated Lp(a) in patients with ASCVD remains underdiagnosed worldwide, despite calls by international guidelines (8). Moreover, the European and other guidelines have recommended for Lp(a) to be tested at least once during each adult's lifetime (8, 16). Current recommendations for hyper-Lp(a) includes adopting healthy lifestyle modifications, and/or pharmacological treatment to target these modifiable cardiovascular risk factors, and intensifying LDL-C targets (6–8, 16–19). Thirdly, our study showed that the lifestyle factors of smoking and good dietary habits were suboptimal in a significant number of patients.

Our observations, that a significant proportion of patients had previously undiagnosed T2DM, hypertension and hypercholesterolemia, supports the purpose of national efforts in population screening, with the aim for earlier diagnosis and management plans to prevent ASCVD events and mortality (20). This has implications in reduction of healthcare costs, hospitalisations, and burden of disease (20). The National Population Health Survey reported that approximately half of all residents with hypercholesterolemia (54.5%) and hypertension (52.4%) diagnosed from 2019 to 2020 were newly diagnosed (9). This exceptionally high prevalence of newly diagnosed hypertension and hypercholesterolemia likely reflects a delayed diagnosis of patients who were unaware of these comorbidities and were diagnosed as a result of the recent increase in population health screening efforts.

The underdiagnosis of major cardiovascular comorbidities have also been identified as a suboptimal medical care in other real-world studies (21, 22). In a United Kingdom study of 466 patients, 51% of patients with IHD did not have any prior cholesterol measurement done (21). In another study of 142,042 patients, it is estimated that at least half of the patients with hypertension are unaware that they have hypertension. Reasons for these include the lack of patients' awareness to undergo health screening and possibly inaccurate blood pressure measurements leading to underdiagnosis (15). Although we did not formally quantify this in our study, our experience during study recruitment revealed similar notions. Reasons provided by patients included that they were unaware of the need to undergo health screening for decades because they felt well. Many were also unaware about the national screening programme available at 40 years old, or earlier in presence of additional risk factors or family history, as per Ministry of Health clinical practice guidelines (23, 24). Other possible reasons included missed opportunities when they went to see a doctor for unrelated conditions. This reduced awareness may also occur at the healthcare system level. A population based study of 52,856 individuals in Norway showed that individuals with undiagnosed diabetes had a less favourable cardiovascular risk profile compared with individuals with known diabetes, which highlights the importance of improving detection strategies (25).

The National Nutrition Survey reported that Singaporean residents on average ate 1 serve of fruit and 2 serves of vegetables daily (26). However, our study population group fared worse, with only ∼10% meeting daily fruit recommendations and ∼20%–30% meeting vegetable recommendations. About 33% of our study population consumed regular wholegrains, compared to ∼6% of residents nationally (26). In general, more than half of the patients with diet history data were not meeting dietary recommendations. Smoking is another lifestyle risk factor that deserves more attention in these patients at very high risk for ASCVD. Compared with other risk factors such as dietary habits, physical activity, alcohol consumption, and obesity, it is smoking that had the strongest association with mortality (27). Importantly, the goal should be for complete smoking cessation, because smoking cessation, not smoking reduction, is the factor that reduces incidence of ASCVD (28). Singapore's “I Quit” national smoking cessation programme, introduced in 2011, reduced the smoking prevalence from 11.8% (year 2017) to 10.1% (year 2020) (29). With the aim to reduce the smoking rate to 5% by 2035, a new smoking cessation programme to subsidise nicotine replacement therapy alongside counselling in public health institutions will be organised by the Health Promotion Board (29). Singapore has also progressively increased the minimum legal age for smoking to 21 years old in 2021 (29). While this may not impact this study group, it has reduced smoking among younger adults aged 18–29 years, from 9.8% in year 2017 to 8.8% in year 2020 (29). The prevalence of active smoking in our study group of IHD patients was particularly high at 28.3%, more so in the newly diagnosed IHD group (34.1%) compared to the known IHD group (23.2%). Since the prevalence of active smoking in our study group was more than double the reported national smoking prevalence of 10.1% (29), this suggests that smoking cessation programmes targeting these patient populations would potentially be of cost benefit.

The limitation of our study was that this was a cross-sectional analysis. Our study was initiated about 5–6 months after COVID-19 outbreak and after a 1-month nationwide lockdown from April to May 2020. This movement restriction and the fear of the rise in cases (30) could have affected our residents' diet, lifestyle, and potentially delayed health screening and doctor visits too, as demonstrated by studies in Hong Kong and Singapore (31–33). A local study showed that doctor visits decreased by 30% and the probability of diagnosing chronic diseases decreased by 19% in that year of COVID-19 outbreak (31). While it is possible that the COVID-19 movement restrictions could have affected these lifestyle and dietary choices of our study participants, the high prevalence of the lifestyle choices and presence of previously undiagnosed metabolic comorbidities is of concern. It is unlikely to fully be explained by restrictions imposed by COVID-19 pandemic alone. Unavailable information included exercise, although this was likely affected by COVID-19 pandemic, and certain dietary fields. For example, we did not specifically identify fibre from other sources such as lentils. However, lentils and beans contribute far less fibre compared to fruits, vegetables and wholegrains, as they are mainly taken in larger quantities by vegetarians (34), whom comprise only 1.6% of our study population. The strengths of our study are that this study showed potential areas for identification and control of risk factors in patients at particularly high risk for ASCVD and requiring hospitalisation. The findings of our study could be helpful to inform future studies and national efforts in prevention of ASCVD. We also analysed a broad range of risk factors for ASCVD, including Lp(a), which was not previously reported in IHD patients requiring hospital admission in Singapore. Because Lp(a) levels are dependent on isoform size, our study had the advantage of uniformly measuring Lp(a) using an isoform-insensitive assay in molar concentrations (6).

## Conclusion

Our study showed that there was a high prevalence of previously undiagnosed chronic metabolic diseases among patients with newly diagnosed IHD, as well as high smoking prevalence in patients with known and newly diagnosed IHD. Increased detection efforts are necessary to diagnose chronic metabolic diseases (hypertension, hypercholesterolemia, T2DM) and elevated Lp(a), especially among patients at high risk for IHD. A heart healthy diet and smoking cessation should be reinforced in patients with IHD.

## Data Availability

The original contributions presented in the study are included in the article, further inquiries can be directed to the corresponding author.
